# Evaluation of the genetic structure of indigenous Okinawa Agu pigs using microsatellite markers

**DOI:** 10.5713/ajas.19.0034

**Published:** 2019-05-28

**Authors:** Shihei Touma, Aisaku Arakawa, Takuro Oikawa

**Affiliations:** 1 Okinawa Prefectural Livestock and Grassland Research Center, Nakijin, Okinawa 905-0426, Japan; 2 Institute of Livestock and Grassland Science, National Agriculture and Food Research Organization (NARO), Tsukuba, Ibaraki 305-0901, Japan; 3 Faculty of Agriculture, University of the Ryukyus, Nishihara, Okinawa 903-0213, Japan

**Keywords:** Agu Pig, Genetic Structure, Genetic Diversity, Indigenous Okinawa Pig, Microsatellite Marker

## Abstract

**Objective:**

Agu pigs are indigenous to the Okinawa prefecture, which is the southernmost region of Japan. Agu pigs were exposed to a genetic bottleneck during the 20th century, due to the introduction of European pig breeds. The objective of this study was to elucidate the genetic structure of Agu pigs and to determine their relationships with those of five European breeds, two Chinese breeds and Ryukyu wild boar using microsatellite markers.

**Methods:**

A total of 203 DNA samples from 8 pig breeds were used in this study. Genotyping was performed using 21 microsatellite markers distributed across 17 chromosomes.

**Results:**

Numbers of effective alleles in Agu pigs were fewer than in European breeds and Ryukyu wild boar. Among domestic pigs, Agu pigs had the lowest heterozygosity (0.423) and highest inbreeding coefficient (F_IS_ = 0.202), indicating a severe loss of heterozygosity in Agu pigs possibly due to inbreeding. Neighbor-joining tree analysis was performed based on Reynolds’ genetic distances, which clustered Agu pigs with Duroc pigs. However, principal component analysis revealed a unique genetic position of the Agu pig, and the second principal component separated Agu pigs from all other breeds. Structure analysis with the optimal assumption of seven groups (K = 7) indicated that Agu pigs form an independent cluster from the other breeds. In addition, high and significant F_ST_ values (0.235 to 0.413) were identified between Agu pigs and the other breeds.

**Conclusion:**

This study revealed a substantial loss of genetic diversity among Agu pigs due to inbreeding. Our data also suggest that Agu pigs have a distinctive genetic structure, although gene flows from European breeds were observed.

## INTRODUCTION

Agu pigs are the only indigenous pig species in Japan that are raised in the Okinawa prefecture, southern Japan. Morphological appearances of Agu pigs are characterized by small sizes (approximately 100 kg at maturity), black coarse hair, semi-erect ears and sagging abdomens. Traditionally, Agu pigs were a typical source of food in Okinawa. But to improve their small body sizes and slow growth, Berkshire and Yorkshire pigs were introduced into Okinawa in 1904. Moreover, after World War II, more productive and economical European breeds were introduced and farmed. These included the breeds Landrace, Large White, and Duroc. As a consequence, Agu pigs faced imminent extinction, with dramatic decreases in the numbers of pure individuals from 104,321 animals in 1899 to 30 in 1980 [1,2]. In the 1980s, the local government collected 18 animals and started a conservation program with several enthusiasts.

It is generally believed that the ancestors of Agu pigs were introduced from the Asian continent, probably from China, in the latter half of the 14th century when the ancient Kingdom of Okinawa began to trade with China [2]. In support of this assertion, phylogenetic analyses using mitochondrial DNA (mtDNA) control regions revealed that the haplotypes of Agu pigs were clustered into the Asian clade [3–5]. However, mtDNA provide only maternal genetic information and does not reflect the history of inheritance as shown by nuclear genomes, which carry male-mediated gene flows. Thus, genetic structures of Agu pigs currently remain unclear. Recently, pork from this breed has again become popular among consumers owing to its superior meat quality, with high intramuscular fat contents and high juiciness [6]. Agu pigs have also been recognized as a valuable genetic resource, and the desire to construct robust conservation schemes for Agu pigs necessitates an understanding of their genetic structure. Thus, we evaluated genetic diversity and phylogenetic relationships among Agu pigs, Chinese and European breeds and Ryukyu wild boar using 21 microsatellite markers.

## MATERIALS AND METHODS

### Animals and DNA sample collection

Tissue samples were collected according to the guidelines for proper conduct of animal experiments of the Science Council of Japan. Tissue samples from 34 Agu pigs were collected from 26 farms on Okinawa and Ishigaki islands. Because pedigree information is incomplete at many Agu pig farms, we collected samples based on interviews with farmers and using pedigree information to ensure that samples were as unrelated as possible. DNA samples from 31 Landrace, 30 Large White, 31 Duroc, 32 Berkshire, 11 Jinhua, and 14 Meishan pigs were collected from several farms in Japan. Twenty DNA samples were collected from Ryukyu wild boars (*Sus scrofa riukiuanus*) on Okinawa, Ishigaki and Iriomote islands. Samples from European breeds were collected from unrelated pigs. Jinhua and Meishan pig samples were collected from an inbred strain because the founding population was small. DNA was extracted using conventional phenol/chloroform techniques.

### Microsatellite genotyping

Genotyping was performed using 21 microsatellite markers distributed across 17 chromosomes; these markers were selected based on informativeness and readability (Supplementary Table S1). Microsatellite fragments were amplified using polymerase chain reactions (PCR) with 42 ng samples of pig genomic DNA and fluorophore-labelled primers. The PCR were performed in final volumes of 15 μL containing 1.5 μL of 10× PCR buffer, 0.9 μL of 25 mM MgCl_2_, 1.5 μL of 8 mM dNTP, 3 pmol of each primer and 0.4 units of Taq DNA polymerase (Applied Biosystems, Foster City, CA, USA). Thermal cycling was performed using a GeneAmp PCR System 9600 (Applied Biosystems, USA) with an initial denaturation for 9 min at 94°C, followed by 40 cycles of 30 s at 94°C, 30 s at annealing temperature (Supplementary Table S1) and 30 s at 72°C and then a final extension step at 72°C for 5 min. To genotype samples, PCR products were electrophoresed using an ABI 3130xl DNA sequencer (Applied Biosystems, USA). Allele sizes were then determined using Gene-Mapper software (Version 3.7, Applied Biosystems, USA).

### Data analyses

Numbers of alleles (N_A_), effective numbers of alleles (N_E_), observed heterozygosity (H_O_), and expected heterozygosity (H_E_) were calculated using GenAlEx version 6.5 software [7]. Pairwise F_ST_ and inbreeding coefficients (F_IS_) were calculated using FSTAT version 2.9.3 [8]. Pairwise comparisons of F_ST_ values were performed using Bonferroni corrections. Deviations from the Hardy–Weinberg equilibrium (HWE) were calculated using GENEPOP program [9]. Polymorphic information contents (PIC) were calculated and allele diversities at each locus were determined using CERVUS version3.0 [10]. Reynolds’ genetic distances [11] were calculated and a phylogenetic tree was constructed via Neighbor-joining tree analysis (NJ) clustering using Populations version 1.2.32 software [12]. Robustness of tree topology was tested using the bootstrap method with 1,000 repetitions. The genetic distances between individuals were determined as −ln(proportions of shared alleles) using a microsatellite analyzer [13] and were used to construct a NJ tree of 203 individuals from 8 pig breeds using MEGA 7.0 [14]. Principal component analysis (PCA) of gene frequencies were performed using the PCAGEN program [15] and Bayesian clustering was performed using STRUCTURE version 2.3.4 [16] to infer population structures and degrees of admixture. Numbers of assumed clusters (K) were estimated by performing 10 independent runs for each K value from 1 to 10 with a burn-in period of 100,000 iterations followed by 100,000 Markov Chain Monte Carlo iterations. The optimal K value was estimated according to the Evanno ΔK method using STRUCTURE HARVESTER 0.6.94 application [17]. The results of STRUCTURE analysis were averaged using CLUMPAK 1.1 [18].

## RESULTS

Polymorphisms of 21 microsatellite loci in 8 breeds are shown in Supplementary Table S2. In accordance with FAO guidelines (N_A_>4 per locus) for population diversity analyses (http://www.fao.org/3/a-aq569e.pdf, Secondary Guidelines MoDAD), N_A_ ranged from 8 (SW72, SW813) to 23 (SW1119). Moreover, all markers apart from SW1437 had PIC of greater than 0.5, indicating that they are highly informative [19].

Genetic diversities of Agu pigs, five European and two Chinese pig breeds and Ryukyu wild boar are shown in Table 1. N_A_ ranged from 2.91 in Jinhua pigs to 6.38 in Landrace pigs. N_E_ were lower in Agu pigs than in European breeds and Ryukyu wild boar. With the exception of Ryukyu wild boar, Agu pigs had the lowest H_O_ (0.423) and the highest of F_IS_ (0.202) values. Numbers of loci that deviated from the HWE in each breed ranged from 1 (Duroc and Jinhua) to 17 (Ryukyu wild boar), whereas 10 loci showed significant deviations from the HWE in Agu pigs (p<0.05).

To evaluate genetic differentiation between Agu pigs and other breeds, we calculated Reynolds’ genetic distances and F_ST_ values (Table 2). The breed with the closest genetic distance to Agu pigs was the Landrace pig (0.251), and the farthest genetic relationship was with Meishan pigs (0.525). Genetic distances between Agu pigs and European pig breeds were shorter (0.251 to 0.280) than those between Agu pigs and Asian breeds (Jinhua, Meishan, and Ryukyu wild boar; 0.407 to 0.525). Moreover, genetic distances were shorter between European pig breeds (0.102 to 0.251) than between Asian breeds (0.369 to 0.392). Pairwise F_ST_ values between Agu pigs and the other breeds (0.235 to 0.413) were significant (p<0.001), falling into the range of *great* to *very great* genetic differentiation [20].

**Figure 1:**
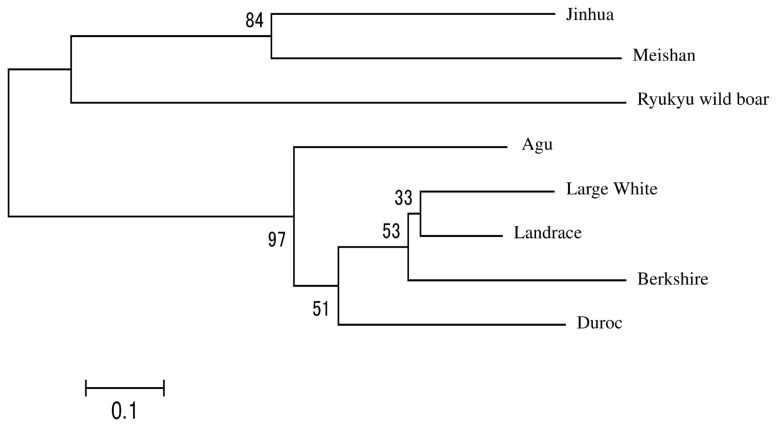
Neighbor-joining tree based on Reynolds’ genetic distance from eight breeds. The numbers at the nodes are bootstrap support values in 1,000 replicates.

In Figure 1, we present a NJ tree that was constructed using Reynolds’ genetic distances to evaluate phylogenetic relationships between Agu pigs and the other breeds. The eight breeds were divided into three clades and the Agu pig was clustered with Duroc pigs. Landrace and Large White and Berkshire pigs formed the second clade, and the Asian breeds formed the remaining clade.

**Figure 2:**
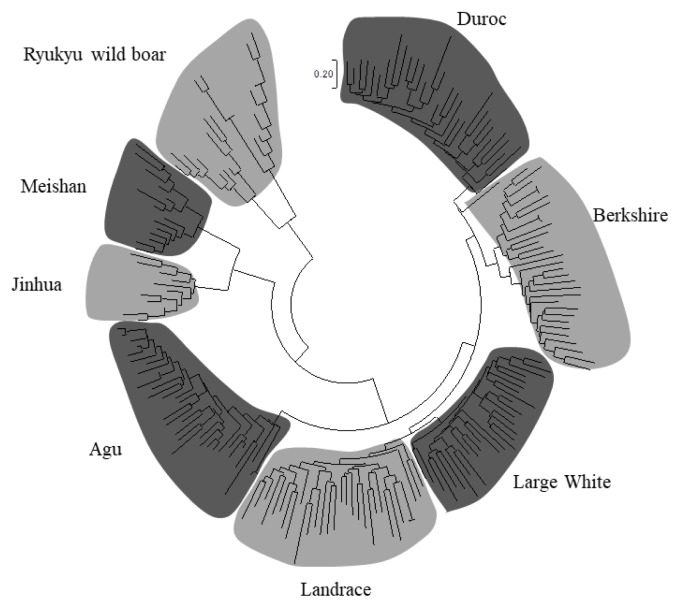
The neighbor-joining tree constructed based on −ln (proportion of share allele) distance among 203 individuals from eight breeds.

We constructed a NJ tree of 203 individuals based on −ln (proportions of shared alleles) distances and determined whether Agu individuals can be distinguished from individuals of other breeds (Figure 2). In these analyses, all individuals were classified into their breed clusters, and 34 Agu individuals formed a single clade without mixing with other breeds.

**Figure 3:**
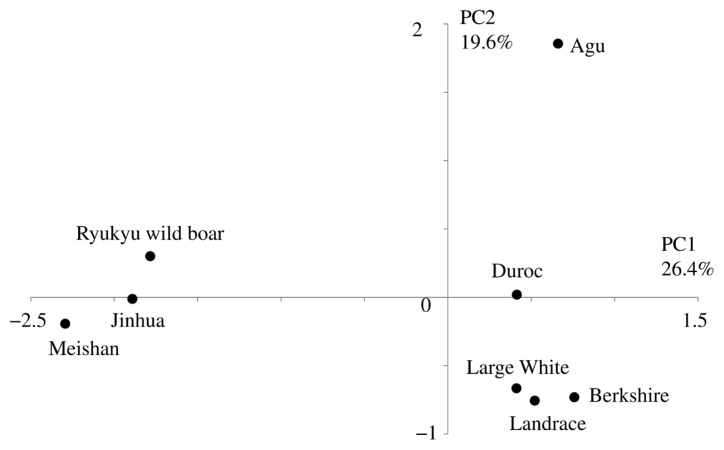
Result of the principal component analysis based on allele frequency of 21 microsatellites. The first and second principal components accounted for 26.4% and 19.6% of the total variance, respectively.

In PCA based on allele frequencies of the 21 microsatellite markers (Figure 3), the first (PC1) and second (PC2) principal components represented 26.4% and 19.6% of the total variation, respectively. This analysis divided the eight breeds into three clusters as follows: cluster I (Asian), Meishan, Jinhua, and Ryukyu wild boar; cluster II (European), Landrace, Large White, Duroc, and Berkshire and cluster III, Agu. In particular, PC1 separated Asian and European breeds and PC2 separated Agu pigs from other breeds.

**Figure 4:**
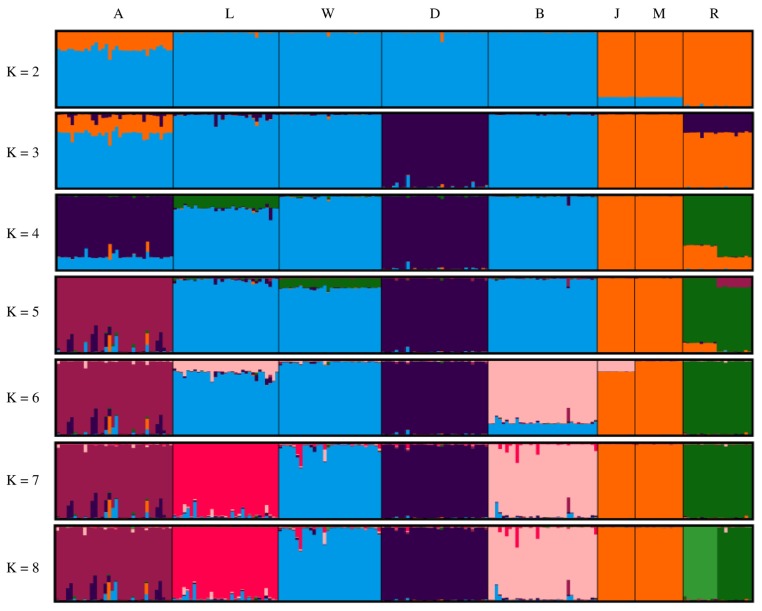
Population structures of eight breeds. Single vertical bars correspond to individual animals, and K indicates the number of assumed clusters. A, Agu; L, Landrace; W, Large white; D, Duroc; B, Berkshire; J, Jinhua; M, Meishan; R, Rykyu wild boar.

Figure 4 shows the results of population structure analysis using STRUCTURE software. Asian and European clusters were generated at K = 2 and Agu pigs were included in both clusters. At K = 5, Agu pigs were clearly separated from the other breeds. Calculations using the Evanno method gave an optimal K value of 7 (Supplementary Figure S1), and population structure analysis at this level generated distinct clusters for all breeds but the Chinese breeds. Finally, Ryukyu wild boar was subdivided into two populations in analysis with K = 8.

## DISCUSSION

### Genetic diversity within Agu pigs

In this study, genetic structures of Agu pigs were investigated using analyses of 21 microsatellite markers in 203 animals from 8 pig breeds. Parameters of genetic diversity among European breeds were within previously reported ranges (Table 1) [21–23], although among Chinese breeds, the H_O_ value of Meishan pigs was lower than that shown in previous studies [23,24]. N_A_ and N_E_ values in Jinhua pigs were lower than in previous studies [25,26], whereas the corresponding H_O_ value was higher. Agu pigs had lower N_E_ than European breeds and Ryukyu wild boar. Among domestic breeds, Agu pigs had the lowest H_O_ value and the highest F_IS_ value, and 10 of 21 markers deviated significantly from the HWE in Agu pigs. These results indicate severe loss of heterozygosity in Agu pig populations, likely resulting from inbreeding. Hence, following replacement with European breeds, low numbers of Agu individuals in a small population likely favored inbreeding. Consequently, Agu pigs had small litter sizes (total number born, 4.8) [27] and low conception rates (20%) [28]. In contrast with Agu pigs, Meishan and Jinhua pigs had low F_IS_ values, likely reflecting breeding strategies that avoid mating between close relatives.

### Genetic influences from European pig breeds in Agu pigs

It is assumed that Japanese Agu pigs originated from the first introduction of pigs from the Asian continent. A previous study of mtDNA control regions by Touma et al [5] revealed that most Agu individuals have East Asian haplotypes, whereas only 14% of Agu pigs carried European haplotypes. These data are evidence of gene flows from European breeds into Agu pig populations. In our studies of nuclear DNA, phylogenetic tree analysis and assessments of Reynolds’ genetic distances revealed shorter genetic distances between Agu pigs and European breeds than between Agu and Chinese breeds (Table 2; Figure 1). Moreover, genetic structure analysis (Figure 4) with K = 2 showed that Agu pigs are a genetic admixture of European and Asian clusters, with predominance of the European cluster. Thus, whereas the nuclear genomes of Agu pigs were influenced by European breeds, Asian mtDNA haplotypes remain predominant. These results suggest that the European breeds that were introduced into Okinawa after 1904 were often used as sires for breeding with Agu females.

### Formation of the current genetic structure of Agu pigs

Taken with a previous study of mtDNA [5], our analyses of nuclear DNA suggest that Agu pig populations have maternal Asian origins, but that strong nuclear genetic influences were imposed from European breeds after 1904. Nonetheless, PCA analysis showed that Agu pigs are genetically unique among European and Asian breeds (Figure 3). Our optimal structure analysis at K = 7, as determined using the Evanno ΔK method, also showed that Agu pigs form an independent cluster from the other breeds (Figure 4). In agreement, population differentiation values (F_ST_) differed greatly between Agu pigs and European and Asian breeds (p<0.001; Table 2), confirming that Agu pigs are genetically distinct. Perhaps the bottleneck effect contributed to this distinctive genetic structure by promoting genetic drift. In addition, Agu pigs were isolated from other pig breeds by the conservation program from the 1980’s, likely enhancing genetic differences. In our NJ tree, which was based on proportions of shared allele distances, 34 Agu individuals were assigned to a single group that was not mixed with other breeds (Figure 2). These data suggest little gene flow between Agu pigs and other breeds in recent decades. However, we have not eliminated the possibility that other breeds contributed to the genetic structure of Agu pigs.

Ryukyu wild boar (*Sus scrofa riukiuanus*) has inhabited the Okinawa islands since the Pleistocene period [29]. In a previous study of mtDNA control regions, no Ryukyu wild boar haplotypes were found in an Agu pig population [5]. Our nuclear DNA analyses also revealed genetic distinctions between Ryukyu wild boar and Agu pigs (Table 2; Figures 1–4), suggesting little genetic influence from Ryukyu wild boar.

Structure analysis at K = 8 subdivided Ryukyu wild boar into two subpopulations (Figure 4), and one of these included individuals from Okinawa Island, whereas the other subpopulation was from Ishigaki and Iriomote islands. Because these subpopulations are separated by sea, the resulting geographic impossibility of gene flows likely contributed to differentiation. Moreover, Ryukyu wild boar had the highest F_IS_ value (0.481) in this study (Table 1), and this may be a consequence of the Wahlund effect [30], which is caused by sampling from subpopulations. The low genetic diversity of this small island population may also have contributed to this high F_IS_ value [24].

## CONCLUSION

We analyzed nuclear DNA using microsatellite markers and revealed a substantial loss of genetic diversity among Agu pigs due to inbreeding. Our data also demonstrated the unique genetic position of Agu pigs, albeit with nuclear gene flow from European breeds. The present findings regarding the genetic structure and diversity of this indigenous pig population will be useful to improve the conservation program for Agu pigs. Currently, high-density single nucleotide polymorphism (SNP) panels, such as the Illumina Porcine SNP 60K BeadChip, permit a more comprehensive genome-wide investigation of genetic structure and genetic diversity than microsatellite markers. To confirm our results, further investigations are needed using SNP arrays.

## Supplementary Data







## Figures and Tables

**Table 1:** Genetic diversity parameters in eight breeds

Breed	N	N_A_	N_E_	H_O_	H_E_	F_IS_	DHWE
Agu	34	5.10	2.31	0.423	0.531	0.202	10
Landrace	31	6.38	3.41	0.608	0.672	0.096	4
Large White	30	5.24	2.87	0.578	0.621	0.069	2
Duroc	31	4.57	2.73	0.568	0.598	0.050	1
Berkshire	32	5.05	2.92	0.561	0.618	0.093	2
Jinhua	11	2.91	2.17	0.566	0.530	−0.067	1
Meishan	14	3.29	2.29	0.466	0.524	0.111	8
Ryukyu wild boar	20	6.10	3.40	0.347	0.668	0.481	17

N_A_, Numbers of alleles; N_E_, effective numbers of alleles; H_O_, observed heterozygosity; H_E_, expected heterozygosity; F_IS_, inbreeding coefficients; DHWE, numbers of loci deviating from Hardy–Weinberg equilibrium (DHWE) at p<0.05 among eight breeds.

**Table 2:** Reynolds’ genetic distance (below diagonal) and pairwise F_ST_ estimates (above diagonal)

Items	A	L	W	D	B	J	M	R
Agu (A)	-	0.235***	0.244***	0.256***	0.256***	0.390***	0.413***	0.336***
Landrace (L)	0.251	-	0.108***	0.165***	0.147***	0.311***	0.329***	0.274***
Large White (W)	0.256	0.102	-	0.227***	0.179***	0.331***	0.342***	0.291***
Duroc (D)	0.280	0.177	0.240	-	0.230***	0.354***	0.363***	0.319***
Berkshire (B)	0.274	0.160	0.192	0.251	-	0.359***	0.371***	0.308***
Jinhua (J)	0.478	0.361	0.372	0.415	0.434	-	0.325***	0.318***
Meishan (M)	0.525	0.397	0.408	0.442	0.455	0.369	-	0.327***
Ryukyu wild boar (R)	0.407	0.321	0.332	0.385	0.371	0.378	0.392	-

*** p<0.001.
