# Supplementary material for: Evaluation of the genetic structure of indigenous Okinawa Agu pigs using microsatellite markers
Source: Asian-Australas J Anim Sci. 2019 May 28;33(2):212–8. doi: 10.5713/ajas.19.0034 (PMC6946958; doi:10.5713/ajas.19.0034)
Supplement: Supplementary file 1 [file ajas-19-0034-suppl1.pdf]

Supplementary Table 1. Information of microsatellite markers used in the present study

Microsatellite markers	Chromosome	Forward Primer (5'-3')	Reverse Primer (5'-3')	Annealing Temperature(°C)	Refference
SW745	1	CTGAGTCTTCTGGGAACTTTTC	ACAGGGCTGGTAGTGTCCC	58	Rohrer et al. (1994)
SW1828 †	1	AATGCATTGTCTTCATTCAACC	TTAACCGGGGCACTTGTG	55	Alexander et al. (1996)
S0226†	2	GCACTTTTAACTTTCATGATACTCC	GGTTAAACTTTTNCCCCAATACA	55	Robic et al. (1994)
SW240 †	2	AGAAATTAGTGCCTCAAATTGG	AAACCATTAAGTCCCTAGCAAA	55	Rohrer et al. (1994)
SW2429	3	TCTTTTTAGGGTGGAGGATGG	CATGTCCCCTATGAACTCTGTG	58	Alexander et al. (1996)
SW72 †	3	ATCAGAACAGTGCGCCGT	TTTGAAAATGGGGTGTTTCC	55	Rohrer et al. (1994)
SWR153	4	ATTTCTTCTTCTTCTTCTTTGTGTG	CTGGGAAGCTCCATATTCCA	58	Alexander et al. (1996)
SWR1526	5	CGGTGGCTACAGATAACAATAC	ATCCGATTCAACCCCTAGC	62	Alexander et al. (1996)
SW1067 †	6	TGCTGGCCAGTGACTCTG	CCGGGGGATTAAACAAAAAG	60	Rohrer et al. (1994)
SW1437	7	TGCTGTATTATACAACACCCGC	TGCCTACTTTATCCTCTGAGGC	58	Smith et al. (1995)
SW933	8	ACATATACTTCCGACAGCCCC	AAGAGCTTGGTGAATTGAGAGC	60	Rohrer et al. (1994)
S0178 †	8	TAGCCTGGGAACCTCCACACGCTG	GGCACCAGGAATCTGCAATCCAGT	60	Rohrer et al. (1996)
SWR1848	9	AAGGGGAAAATCCCTCAAAG	TTTCTATGCA AATTTTTCCGTG	60	Alexander et al. (1996)
SW443	10	ACAAAGGCCAAGCCACATAC	TCACCAGGTTTCTGGGTTTC	60	Rohrer et al. (1994)
SW1415	11	AATGGCTAAGGAACTTCTGCC	CTAGTTATTGCCTGGTGGCC	60	Alexander et al. (1996)
SW957	12	AGGAAGTGAGCTCAGAAAGTGC	ATGGACAAGCTTGGTTTTCC	58	Rohrer et al. (1994)
SWR1941 †	13	AGAAAGCAATTTGATTTGCATAATC	ACAAGGACCTACTGTATAGCACAGG	55	Alexander et al. (1996)
SW1027	14	AGCAACCTGAGCCACAGTG	GGAACTTCCACACGCCAC	60	Rohrer et al. (1994)
SW1119	15	CAACCTCAAAAATGGAGAAAGG	GTTCTTGCGGTGTTTGGC	60	Rohrer et al. (1994)
SW813	16	AGTTGATTTAAAATGTTGTGCCA	AATATTTCAAAAAAAGGAATGCG	58	Rohrer et al. (1994)
SW24 †	17	CTTTGGGTGGAGTGTGTGC	ATCCAAATGCTGCAAGCG	55	Rohrer et al. (1994)

† These 8 markers are recommended by the Domestic Animal Diversity Information System of the Food and Agriculture Organization of the United Nations (FAO 2004).

1. Alexander LJ, Rohrer GA, Beattie CW. Cloning and characterization of 414 polymorphic porcine microsatellites. Anim Genet 1996;27:137-48.

2. Ellegren H, Chowdhary BP, Johansson M, et al. A primary linkage map of the porcine genome reveals a low rate of genetic recombination. Genetics 1994;137:1089-100.

3. Robic A, Dalens M, Woloszyn N, Milan D, Riquet J, Gellin J. Isolation of 28 new porcine microsatellites revealing polymorphism. Mamm Genome 1994;5:580-3.

4. Rohrer GA, Alexander LJ, Keele JW, Smith TP, Beattie CW. A microsatellite linkage map of the porcine genome. Genetics 1994;136:231-45.

5. Rohrer GA, Alexander LJ, Hu Z, Smith TP, Keele JW, Beattie CW. A comprehensive map of the porcine genome. Genome Res 1996;6:371-91.

6. Smith TPL, Rohrer GA, Alexander LJ, et al. Directed integration of the physical and genetic linkage maps of swine chromosome 7 reveals that the SLA spans the centromere. Genome Res 1995;5:259-71.
