# Supplementary material for: Evaluation of the genetic structure of indigenous Okinawa Agu pigs using microsatellite markers
Source: Asian-Australas J Anim Sci. 2019 May 28;33(2):212–8. doi: 10.5713/ajas.19.0034 (PMC6946958; doi:10.5713/ajas.19.0034)
Supplement: Supplementary file 2 [file ajas-19-0034-suppl2.pdf]

Supplementary Table 2. Numbers of alleles (N_A), effective numbers of alleles (N_E), observed (H_O) and expected (H_E) heterozygosities, polymorphism information content (PIC) of 21 microsatellite loci in eight breeds

Locus	N_A	N_E	H_O	H_E	PIC
SW745	16	2.10	0.394	0.778	0.750
SW1828	10	2.44	0.468	0.831	0.808
S0226	12	2.47	0.369	0.759	0.720
SW240	15	3.04	0.604	0.837	0.822
SW2429	10	2.57	0.557	0.836	0.814
SW72	8	2.33	0.460	0.793	0.761
SWR153	12	3.07	0.605	0.773	0.743
SWR1526	18	3.18	0.621	0.827	0.805
SW1067	15	3.20	0.700	0.879	0.865
SWR1437	10	1.73	0.266	0.502	0.481
SW933	12	2.40	0.458	0.737	0.713
S0178	9	2.63	0.564	0.793	0.762
SWR1848	13	2.96	0.596	0.857	0.839
SW443	15	3.14	0.589	0.829	0.809
SW1415	14	2.74	0.493	0.827	0.807
SW957	15	2.50	0.468	0.761	0.745
SWR1941	9	2.63	0.540	0.770	0.733
SW1027	16	3.71	0.610	0.873	0.857
SW1119	23	3.42	0.611	0.775	0.747
SW813	8	2.59	0.369	0.786	0.757
SW24	13	3.16	0.608	0.851	0.832
Mean	13.0	2.76	0.521	0.785	0.770
