# Supplementary figures and images for: Evaluation of the genetic structure of indigenous Okinawa Agu pigs using microsatellite markers
Source: Asian-Australas J Anim Sci. 2019 May 28;33(2):212–8. doi: 10.5713/ajas.19.0034 (PMC6946958; doi:10.5713/ajas.19.0034)

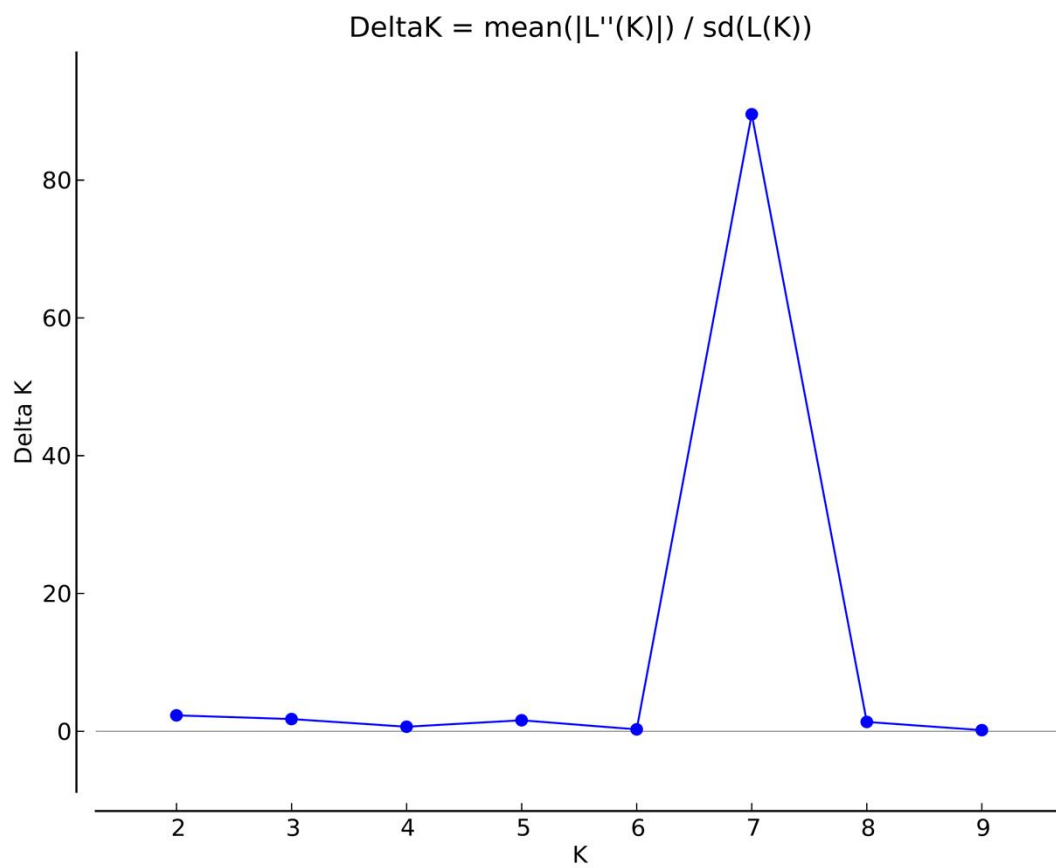

**Supplementary Figure 1.** Plot of Delta K calculated by the method of Evanno et al [17].

Supplement: Supplementary file 3 [file ajas-19-0034-suppl3.pdf]
